# Usefulness of Ankle-Brachial Index Calculated Using Diastolic Blood Pressure and Mean Arterial Pressure in Predicting Overall and Cardiovascular Mortality in Hemodialysis Patients

**DOI:** 10.7150/ijms.50831

**Published:** 2021-01-01

**Authors:** Po-Chao Hsu, Jiun-Chi Huang, Wen-Hsien Lee, Ying-Chih Chen, Pei-Yu Wu, Wei-Chung Tsai, Szu-Chia Chen, Ho-Ming Su

**Affiliations:** 1 Division of Cardiology, Department of Internal Medicine, Kaohsiung Medical University Hospital, Kaohsiung, Taiwan;; 2 Division of Nephrology, Department of Internal Medicine, Kaohsiung Medical University Hospital, Kaohsiung Medical University, Kaohsiung, Taiwan;; 3 Department of Internal Medicine, Kaohsiung Municipal Siaogang Hospital, Kaohsiung, Taiwan;; 4 Faculty of Medicine, College of Medicine, Kaohsiung Medical University, Kaohsiung, Taiwan;; 5 Research Center for Environmental Medicine, Kaohsiung Medical University, Kaohsiung, Taiwan; 6 Regenerative Medicine and Cell Therapy Research Center, Kaohsiung Medical University, Kaohsiung, Taiwan

**Keywords:** ankle-brachial index, systolic blood pressure, diastolic blood pressure, mean arterial pressure, overall mortality, hemodialysis

## Abstract

No study has investigated the predictive ability of ankle-brachial index (ABI) calculated using diastolic blood pressure (DBP) (ABIdbp) and mean arterial pressure (MAP) (ABImap) for overall and cardiovascular (CV) mortality in hemodialysis (HD) patients. Our study was aimed to investigate the issue. Two hundred and seven routine HD patients were enrolled. ABI values were measured by ABI-form device. During the follow-up period (122 months), 124 of the 207 patients (59.0%) died, and 59 deaths due to CV cause. Multivariate analysis showed that low ABIsbp, ABIdbp, and ABImap were all significantly associated with increased overall (*p* ≤ 0.015) and CV mortality (*p* ≤ 0.015) in whole study patients. A subgroup analysis after excluding 37 patients with ABIsbp < 0.9 or > 1.3 found ABIsbp and ABIsbp < 0.9 were not associated with overall and CV mortality. However, ABImap and ABIdbp < 0.87 were significantly associated with overall mortality (*p* ≤ 0.042). Furthermore, ABIdbp and ABIdbp < 0.87 were significantly associated with CV mortality (*p* ≤ 0.030). In conclusion, ABIsbp, ABIdbp, and ABImap were all useful in predicting overall and CV mortality in our HD patients. In the subgroup patients with normal ABIsbp, ABIsbp and ABIsbp < 0.9 were not useful to predict overall and CV mortality. Nevertheless, ABImap and ABIdbp < 0.87 could still predict overall mortality, and ABIdbp and ABIdbp < 0.87 could predict CV mortality. Hence, calculating ABI using DBP and MAP may provide benefit in survival prediction in HD patients, especially in the patients with normal ABIsbp.

## Introduction

Patients undergoing hemodialysis (HD) have a high prevalence of peripheral artery occlusive disease (PAOD), which is associated with chronic renal failure and long-term diabetes 1. PAOD can be diagnosed using the ankle-brachial index (ABI), which is a simple and noninvasive tool. ABI was calculated as the ankle systolic blood pressure (SBP) divided by the brachial SBP. The original rationale of comparing ankle SBP with brachial SBP was first introduced by Winsor T who reported that obstruction of the artery results in pressure drop below the obstructed site 2.Therefore, SBP at various location on the extremities would give an insight to the location and degree of obstruction.

A low ABI has been shown to have a high sensitivity and specificity for the diagnosis of PAOD, and also to be strongly associated with cardiovascular (CV) and overall mortality in HD patients 3,4. However, the presence of arterial media calcification can considerably reduce the sensitivity of the ABI to correctly diagnose PAOD. Therefore, it is important to improve the predictive power of ABI for mortality in HD patients.

A low ABI calculated using systolic blood pressure (SBP) (ABIsbp) has been associated with higher rates of CV and overall mortality in patients with chronic kidney disease 5, ischemic heart disease 6, diabetes 7, and atrial fibrillation 8, and also in those undergoing HD 9. In clinical studies and real-world clinical practice, the ABI is always calculated using SBP. However, diastolic blood pressure (DBP) has been reported to be useful in the prediction of mortality in patients with stroke 10, in the elderly with CV disease 11, in individuals with subclinical atherosclerosis 12, and in patients with chronic kidney disease 13. Moreover, mean arterial pressure (MAP), a relatively steady blood pressure component, has also been associated with mortality in normotensive individuals 14, and in patients with left ventricular dysfunction 15. Our recent study showed that ABIsbp, ABI using MAP (ABImap), and ABI using DBP (ABIdbp) had similar predictive value for PAOD, and ABImap could provide extra benefit in survival prediction for patients enrolled from echocardiographic examination 16. However, no previous study has investigated the ability of ABImap and ABIdbp in predicting overall and CV mortality in HD patients. Accordingly, the aim of this study was to investigate whether ABImap and ABIdbp were useful parameters in the prediction of overall and CV mortality in HD patients, and compare the predictive values of ABIsbp, ABImap, and ABIdbp for overall and CV mortality in these patients.

## Materials and Methods

### Study Patients and Design

This study evaluated all routine HD patients attending one dialysis clinic in a regional hospital in Taiwan. Five patients who refused to undergo ABI-form device examinations were excluded. In addition, four patients with atrial fibrillation, two with bilateral amputations below the knee, and five who had been hospitalized or received antibiotic treatment in the preceding 4 weeks were also excluded. The remaining 207 patients (92 males and 115 females) were enrolled as the study group in December 2006. We acquired informed consents from the patients and conducted our study according to the declaration of Helsinki. All of the enrolled patients underwent HD three times per week. Each HD session lasted 3.5-4.5 hours, and the blood flow and dialysate flow rates were 250-300 mL/min and 500 mL/min, respectively. The study protocol was approved by the Institutional Review Board of Kaohsiung Medical University Hospital. The methods were carried out in accordance with the approved guidelines.

### Assessment of ABI

An ABI-form device was used to measure the ABI values 10-30 minutes before each HD session. The device measured oscillometric blood pressures in the arms and ankles simultaneously, as previously reported 17. Occlusion and monitoring cuffs without blood access were placed tightly around the upper arm and both sides of the lower extremities with the patient in the supine position. ABIsbp was calculated as the lower ankle SBP divided by the brachial SBP. Similarly, ABImap was calculated as the lower ankle MAP divided by the brachial MAP, and ABIdbp was calculated as the lower ankle DBP divided by the brachial DBP. The ABI was measured once in each patient.

### Demographic, Medical and Laboratory Data

Data on age, sex, smoking history (former *vs.* current), and comorbidities were obtained from medical records and patient interviews. The body mass index (BMI) was calculated as weight in kilograms divided by the square of height in meters. Fasting blood samples were obtained from all of the patients within 1 month of enrollment, and laboratory data were obtained using an autoanalyzer (Roche Diagnostics GmbH, D-68298 Mannheim COBAS Integra 400). In addition, information regarding the patients' medications including angiotensin converting enzyme inhibitors, angiotensin II receptor blockers, β-blockers, and calcium channel blockers at enrollment was obtained from medical records.

### Definition of Overall and CV Mortality

Data on overall and CV mortality were collected from the Collaboration Center of Health Information Application (CCHIA), Ministry of Health and Welfare, Executive Yuan, Taiwan, up to December 2019.

### Statistical Analysis

All statistical analyses were performed using SPSS version 22.0 (SPSS, Chicago, IL, USA). Data were expressed as mean ± standard deviation, percentage, or median (25th-75th percentile) for the follow-up period. Differences between groups were analyzed using the Chi-square test for categorical variables, or the independent samples t-test for continuous variables. We created several models to identify the optimum cut-off values of the ABIs to predict overall mortality. The model with the best performance in predicting overall mortality was identified using the Chi-square value, and consisted of ABIdbp < 0.87 and ABImap < 0.92. Significant variables in univariate analysis were entered into multivariate analysis. A Cox proportional hazards model was used to analyze the time to mortality. All tests were two-sided, and the level of significance was defined as *p* < 0.05.

## Results

A total of 207 HD patients were included (92 men and 115 women), with a mean age of 59 ± 13 years. The prevalence rates of ABIsbp < 0.9, ABIdbp < 0.87, and ABImap < 0.92 were 12.9%, 21.9%, and 17.1%, respectively. The median follow-up period was 122 months (25th-75th percentile: 58-157 months) for all patients. During the follow-up period, 124 of the 207 patients (59.0%) died due to CV causes (n = 59), malignancy (n = 6), infectious diseases (n = 44), gastrointestinal bleeding (n = 6), and others (n = 9). Table 1 shows comparison of baseline characteristics between patients with and without mortality. Compared to the patients without mortality, patients with mortality had older age, higher percentage of diabetes, higher SBP and triglyceride, lower albumin, and higher usage of calcium channel blockers. Regarding ABI data, the patients with mortality had a lower ABIsbp, ABIdbp, and ABImap.

### Predictors of overall and CV mortality in the univariate analysis in all study patients

The results of univariate Cox proportional hazards regression analysis for overall and CV mortality in all study patients are shown in Table 2. Univariate regression analysis showed that old age, diabetes, high SBP, a low level of albumin, high level of triglycerides, and high usage of calcium channel blockers were associated with increased overall mortality. Regarding associations between ABI data and outcomes, low ABIsbp, low ABIdbp, and low ABImap (all *p* < 0.001) were associated with increased overall mortality. In addition, old age, diabetes, a low level of albumin, high level of triglycerides, and high usage of calcium channel blockers were associated with increased CV mortality. Regarding associations between ABI data and outcomes, low ABIsbp, low ABIdbp, and low ABImap (all *p* < 0.001) were associated with increased CV mortality.

### Relation of ABI data for overall and CV mortality in the multivariate analysis in all study patients and patients with 0.9 ≤ ABIsbp ≤ 1.3

The results of multivariate Cox proportional hazards regression analysis for overall and CV mortality in all study patients are shown in Table 3, For overall mortality, we adjusted the significant clinical variables in the univariate analysis including age, diabetes, SBP, albumin, triglycerides, and the use of calcium channel blockers. For CV mortality, we adjusted age, diabetes, albumin, and triglycerides. In the multivariate analysis, low ABIsbp (per 1; hazard ratio [HR], 0.188; 95% confidence interval [CI], 0.068 to 0.519, *p* = 0.001), low ABIdbp (per 1; HR, 0.214; 95% CI, 0.062 to 0.744, *p* = 0.015) and low ABImap (per 1; HR, 0.128; 95% CI, 0.036 to 0.461, *p* = 0.002) were all significantly associated with increased overall mortality. In addition, low ABIsbp (per 1; HR, 0.179; 95% CI, 0.045 to 0.713, *p* = 0.015), low ABIdbp (per 1; HR, 0.102; 95% CI, 0.020 to 0.535, *p* = 0.007) and low ABImap (per 1; HR, 0.097; 95% CI, 0.017 to 0.542, *p* = 0.008) were all significantly associated with increased CV mortality.

We further performed a subgroup analysis after excluding 37 patients with ABIsbp < 0.9 or > 1.3. The results of multivariate Cox proportional hazards regression analysis for overall and CV mortality in these 170 patients are shown in Table 3. ABIsbp was not associated with overall and CV mortality in this subgroup analysis. Only low ABImap (per 1; HR, 0.063; 95% CI, 0.004 to 0.903, *p* = 0.042) was significantly associated with increased overall mortality and low ABIdbp (per 1; HR, 0.051; 95% CI, 0.003 to 0.748, *p* = 0.030) was significantly associated with increased CV mortality.

### Relation of ABI data using cutoff value for overall and CV mortality prediction in patients with 0.9 ≤ ABIsbp ≤ 1.3

Because ABIsbp could not predict overall and CV mortality in the subgroup patients with normal ABI, we tried to use cutoff values of ABIsbp, ABIdbp, and ABImap to evaluate the abilities of these different ABI cutoff values for prediction of overall and CV mortality and the results are shown in Table 4. ABIsbp < 0.9 has been considered as the useful cutoff value for prediction of mortality. In this study, we used Chi-square value to select the model with the best performance and finally we found the model using ABImap < 0.92 and ABIdbp < 0.87 had the best performance in predicting mortality. Therefore, ABIsbp < 0.9, ABIdbp < 0.87, and ABImap < 0.92 were used as the cutoff values to predict overall and CV mortality in the subgroup patients with normal ABI. After multivariate analysis, ABIsbp < 0.9 still was not associated with increased overall and CV mortality. However, ABIdbp < 0.87 was a significant predictor of overall and CV mortality.

## Discussion

In our study, multivariate analysis showed that ABIsbp, ABIdbp, and ABImap could predict overall and CV mortality in all HD patients. After excluding patients with abnormal ABIsbp (< 0.9 or > 1.3), ABIsbp and ABIsbp < 0.9 could not predict overall and CV mortality. However, ABImap and ABIdbp < 0.87 could still predict overall mortality, and ABIdbp and ABIdbp < 0.87 could predict CV mortality.

The main strength of this study is the prospective follow-up data of HD patients over a median follow-up period of 122 months, a group known to be at high risk of PAOD. To the best of our knowledge, no previous study has investigated the ability of ABImap and ABIdbp to predict mortality in this population.

The first important finding of this study is that ABIsbp, ABIdbp, and ABImap were all associated with increased overall and CV mortality in whole HD patients. This finding was similar to our previous study which evaluated and compared different ABIs in prediction of long-term overall and CV mortality for patients enrolled from echocardiographic examination 16. In that study, ABIsbp, ABIdbp, and ABImap were all significant predictors of overall and CV mortality after multivariate analysis.

The second important finding of this study is that ABIdbp < 0.87 was associated with increased overall and CV mortality in the subgroup patients with normal ABIsbp. Furthermore, ABIdbp could also predict CV mortality in the subgroup analysis. DBP is a component of pulse pressure and increased aortic stiffness causes an increase in pulse pressure. A low DBP has been reported to result in decreased coronary perfusion during the diastolic phase of the cardiac cycle particularly in patients with coronary heart disease and consequently a very low DBP may cause myoischemia 18,19. Many observational studies have shown an association between a low DBP and a higher rate of CV events, the so-called J-curve 20. A systematic review of 13 studies reported that patients with a DBP < 85 mmHg were associated with an increased risk of CV events 21. In addition, McEvoy et al. demonstrated an association of a low DBP with an increased level of high-sensitivity troponin T and high rates of CV events and all-cause mortality 20. Moreover, Tuomilehto et al. reported that low DBP alone was a significant predictor of CV and non-CV mortality in people aged >50 years (and most commonly in those >70 years 22. Even though DBP is not considered to be the most importance factor in the management of hypertension, both SBP and DBP target values are recorded by clinicians. Controversy also exists regarding the possibility of a J-curve relationship between DBP and outcomes and some studies have reported a higher risk of adverse outcomes with both a high and low DBP 23. This is especially concerning as lower targets in revised hypertension guidelines may lead diastolic hypotension due to over treatment 24. In our study, ABIdbp < 0.87 played an important role in predicting overall and CV mortality in HD patients with normal ABI. In addition, ABIdbp could not only predict overall and CV mortality in whole HD patients, but also predict CV mortality in subgroup patients with normal ABIsbp. The exact mechanism for this finding is unclear. We suggest that in patients undergoing HD with a high burden of CV disease, even in those without evidence of severe coronary heart disease, oxygen delivery may be impaired during HD, which may impair subendocardial contractility. Although ABIsbp had an important predictive ability, ABIsbp and ABIdbp each independently influenced mortality and therefore ABIdbp should not be ignored and even had better predictive value of mortality than ABIsbp in HD patients with normal ABIsbp.

The third important finding of this study is that a low ABImap was associated with an increased risk of overall and CV mortality in whole HD patients. In addition, ABImap could also predict overall mortality in subgroup patients with normal ABIsbp. MAP is defined as the average arterial pressure during one cardiac cycle, systole and diastole, and it is a relatively steady blood pressure component. Perfusion of vital organs requires a minimum MAP of 60 mmHg and end-organ ischemia or infarction can occur if it falls below this level for a prolonged period of time. Furthermore, a significant decrease in MAP can prevent blood from adequately perfusing tissues 25. In this study, ABImap played a vital role in predicting mortality in HD patients, although the exact mechanism of this finding is unclear. A low ABImap value may reflect a lower tissue perfusion pressure somewhere other than in the upper extremities. The relatively low tissue perfusion pressure may result in a poor prognosis in long-term follow-up.

There are also several limitations in our study. First, including patients with both high and low values of ABI could be misleading. Patients with an ABIsbp > 1.3 may have medial arterial calcification and thus have non-compressible arteries and those with an ABIsbp < 0.9 may have arteriosclerotic disease. However, we performed a subgroup analysis in patients with normal ABIsbp for further elucidation. Second. ABI may be not a good marker for PAOD in HD patients and future studies should use alternative methods such as flow wave analysis or Doppler color ultrasound. Third, ABI was only measured once in our HD patients which may cause some variations in measurement of blood pressure. Finally, although the different ABIs have an impact on mortality prediction, the physiological differences among these three ABIs are unknown. Further studies are necessary to address this issue.

In conclusion, we compared different ABIs with regards to predicting survival in HD patients. Multivariate analysis showed that ABIsbp, ABIdbp, and ABImap could predict overall and CV mortality in all study patients. After excluding patients with abnormal ABIsbp (< 0.9 or > 1.3), ABImap and ABIdbp could still predict overall and CV mortality, respectively in the multivariate analysis, but ABIsbp could not. Furthermore, ABIdbp < 0.87 could also predict overall and CV mortality in this subgroup analysis. Hence, calculating ABI using DBP and MAP may provide benefit in survival prediction in HD patients, especially in the subgroup patients with normal ABIsbp.

## Figures and Tables

**Figure 1:**
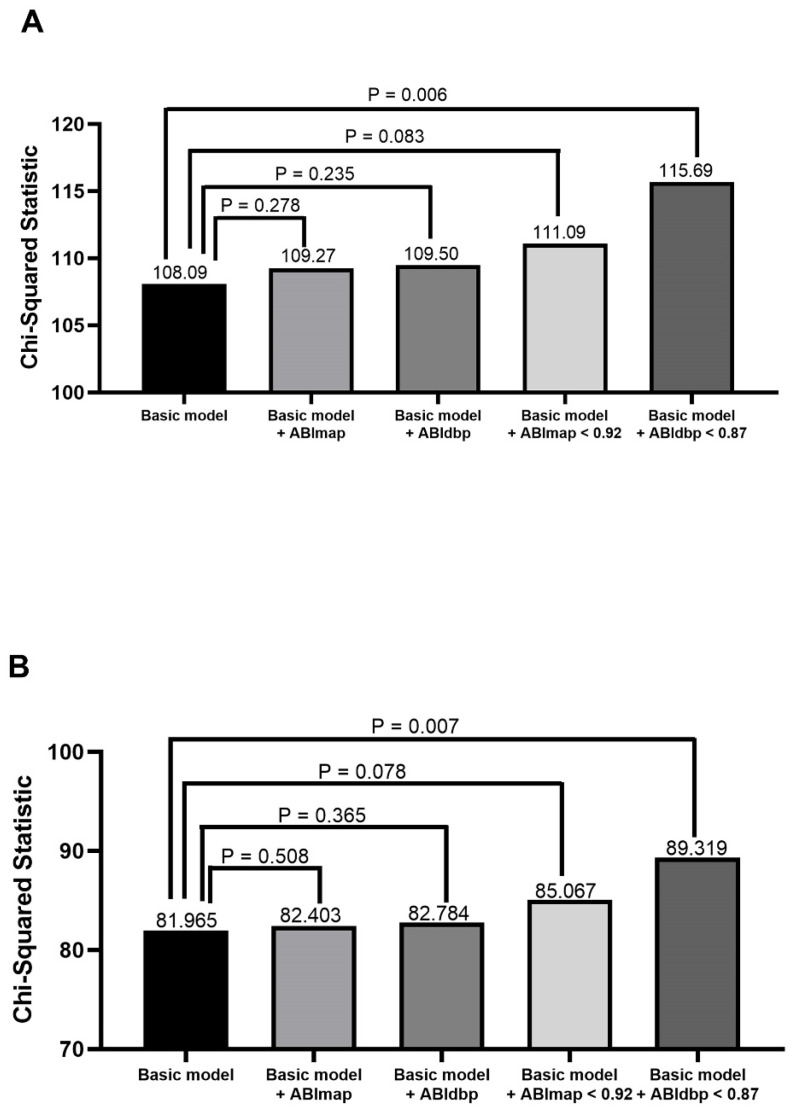
Comparison of the prediction power of addition of ABImap, ABIdbp, ABImap < 0.92, and ABIdbp < 0.87 to a basic model in the prediction of overall mortality in all patients (A) and patients with 0.9 ≤ ABIsbp ≤ 1.3 (B). The variables in the basic model included age, diabetes mellitus, systolic blood pressure, albumin, triglyceride, using of calcium channel blockers, and ABIsbp.

**Figure 2:**
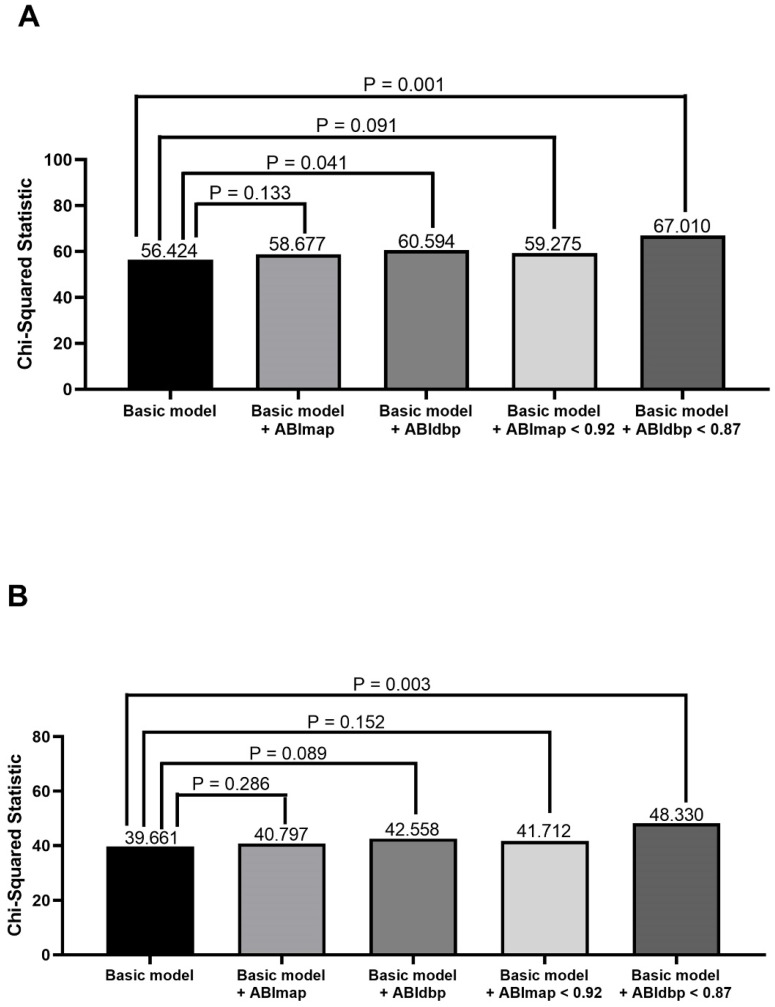
Comparison of the prediction power of addition of ABImap, ABIdbp, ABImap < 0.92, and ABIdbp < 0.87 to a basic model in the prediction of cardiovascular mortality in all patients (A) and patients with 0.9 ≤ ABIsbp ≤ 1.3 (B). The variables in the basic model included age, diabetes mellitus, and ABIsbp.

**Table 1:** Comparison of baseline characteristics between patients with and without mortality

Characteristics	Mortality (-) (n = 83)	Mortality (+) (n = 124)	*p*	All patients (n = 207)
Age (year)	50 ± 11	65 ± 11	<0.001	59 ± 13
Male gender (%)	47	43	0.571	44
Diabetes mellitus (%)	19	52	<0.001	39
Hypertension (%)	66	74	0.274	71
Current smoking (%)	6	10	0.443	8
SBP (mmHg)	140 ± 23	149 ± 25	0.007	145 ± 25
DBP (mmHg)	81 ± 14	79 ± 16	0.315	80 ± 15
Body mass index (kg/m^2^)	23.7 ± 3.6	24.0 ± 3.6	0.631	23.9 ± 3.6
Heart rate (min^-1^)	80 ± 14	80 ± 12	0.725	80 ± 13
Albumin (g/dL)	3.91 ± 0.25	3.78 ± 0.29	0.002	3.83 ± 0.28
Hemoglobin (g/dL)	9.8 ± 1.2	10.0 ± 1.1	0.362	9.9 ± 1.1
Triglyceride (mg/dL)	146 ± 93	190 ± 147	0.009	172 ± 130
Total cholesterol (mg/dL)	184 ± 38	186 ± 44	0.747	185 ± 42
Antihypertensive medications				
ACEI and/or ARB (%)	18	20	0.723	19
β-blocker (%)	17	20	0.682	19
Calcium channel blocker (%)	27	42	0.026	36
ABI data				
ABIsbp	1.14 ± 0.12	1.04 ± 0.19	< 0.001	1.09 ± 0.17
ABIdbp	0.98 ± 0.10	0.91 ± 0.16	< 0.001	0.94 ± 0.14
ABImap	1.06 ± 0.09	0.98 ± 0.15	< 0.001	1.01 ± 0.14

Abbreviations. ABI, ankle-brachial index; ACEI, angiotensin converting enzyme inhibitor; ARB, angiotensin II receptor blocker; DBP, diastolic blood pressure; MAP, mean arterial pressure; SBP, systolic blood pressure.

**Table 2:** Predictors of overall and cardiovascular mortality using Cox proportional hazards model in the univariate analysis in all study patients

Parameters	Overall mortality		Cardiovascular mortality	
	HR (95% CI)	*p*	HR (95% CI)	*p*
Age (per 1 year)	1.070 (1.053-1.087)	< 0.001	1.061 (1.037-1.086)	< 0.001
Male (*vs.* female)	0.872 (0.611-1.244)	0.450	0.921 (0.551-1.540)	0.753
Diabetes mellitus	2.356 (1.651-3.361)	< 0.001	3.243 (1.920-5.478)	< 0.001
Hypertension	1.353 (0.905-2.023)	0.141	1.507 (0.827-2.747)	0.180
Current smoking	1.144 (0.631-1.075)	0.658	1.669 (0.792-3.518)	0.178
SBP (per 1 mmHg)	1.010 (1.003-1.018)	0.007	1.009 (0.998-1.020)	0.106
DBP (per 1 mmHg)	0.993 (0.981-1.005)	0.253	0.990 (0.973-1.007)	0.245
Body mass index (per 1 kg/m^2^)	1.005 (0.956-1.057)	0.852	1.065 (0.991-1.144)	0.087
Heart rate (per 1 min^-1^)	0.997 (0.984-1.011)	0.699	1.000 (0.981-1.020)	0.998
Albumin (per 1 g/dL)	0.326 (0.189-0.562)	< 0.001	0.310 (0.143-0.672)	0.003
Hemoglobin (per 1 g/dL)	1.068 (0.915-1.247)	0.404	1.072 (0.857-1.340)	0.544
Triglyceride (per 1 mg/dL)	1.002 (1.000-1.003)	0.013	1.002 (1.001-1.004)	0.006
Total cholesterol (per 1mg/dL)	1.001 (0.997-1.005)	0.691	1.002 (0.996-1.008)	0.485
Antihypertensive medications				
ACEI and/or ARB use	1.203 (0.775-1.866)	0.410	1.324 (0.715-2.451)	0.371
β-blocker use	1.102 (0.637-1.909)	0.728	1.264 (0.602-2.656)	0.536
CCB use	1.437 (1.004-2.056)	0.047	1.661 (0.994-2.774)	0.053
ABI data				
ABIsbp (per 1)	0.061 (0.023-0.163)	< 0.001	0.048 (0.012-0.197)	< 0.001
ABIdbp (per 1)	0.049 (0.015-0.165)	< 0.001	0.025 (0.005-0.134)	< 0.001
ABImap (per 1)	0.027 (0.008-0.093)	< 0.001	0.018 (0.003-0.103)	< 0.001

**Abbreviations:** CCB, calcium channel blocker; CI, confidence interval; HR, Hazard Ratios. Other abbreviations as table 1.

**Table 3:** Relation of ABI data for overall and CV mortality using Cox proportional hazards model in the multivariate analysis in all study patients and patients with 0.9 ≤ ABIsbp ≤ 1.3

ABI data	All patients		Patients with 0.9 ≤ ABIsbp ≤ 1.3	
	HR (95% CI)	*p*	HR (95% CI)	*p*
**Overall mortality**				
ABIsbp (per 1)	0.188 (0.068-0.519)	0.001	0.130 (0.016-1.063)	0.053
ABIdbp (per 1)	0.214 (0.062-0.744)	0.015	0.059 (0.019-1.080)	0.059
ABImap (per 1)	0.128 (0.036-0.461)	0.002	0.063 (0.004-0.903)	0.042
**CV mortality**				
ABIsbp (per 1)	0.179 (0.045-0.713)	0.015	0.126 (0.006-2.556)	0.177
ABIdbp (per 1)	0.102 (0.020-0.535)	0.007	0.051 (0.003-0.748)	0.030
ABImap (per 1)	0.097 (0.017-0.542)	0.008	0.032 (0.001-1.403)	0.074

**Abbreviations:** CI, confidence interval; CV, cardiovascular; HR, Hazard Ratios. Other abbreviations as table 1. Covariates in the multivariate model for overall mortality included significant clinical variables in the univariate analysis, which consisted of age, diabetes mellitus, systolic blood pressure, albumin, triglyceride, and using of calcium channel blockers. Covariates in the multivariable model for CV mortality included significant clinical variables in the univariate analysis, which consisted of age, diabetes mellitus, albumin, and triglyceride.

**Table 4:** Relation of ABI data using cutoff value for overall and CV mortality prediction in patients with 0.9 ≤ ABIsbp ≤ 1.3 (multivariate analysis)

ABI data	Patients with 0.9 ≤ ABIsbp ≤ 1.3	
	HR (95% CI)	*p*
**Overall mortality**		
ABIsbp < 0.9	-	-
ABIdbp < 0.87	2.176 (1.323-3.578)	0.002
ABImap < 0.92	2.039 (0.941-4.419)	0.071
**CV mortality**		
ABIsbp < 0.9	-	-
ABIdbp < 0.87	3.096 (1.571-6.101)	0.001
ABImap < 0.92	2.399 (0.877-6.558)	0.088

**Abbreviations:** CI, confidence interval; CV, cardiovascular; HR, Hazard Ratios. Other abbreviations as table 1. Covariates in the multivariate model for overall mortality included significant clinical variables in the univariate analysis, which consisted of age, diabetes mellitus, systolic blood pressure, albumin, triglyceride, and using of calcium channel blockers. Covariates in the multivariable model for CV mortality included significant clinical variables in the univariate analysis, which consisted of age, diabetes mellitus, albumin, and triglyceride.

## References

[1] Leskinen Y, Salenius JP, Lehtimaki T (2002). The prevalence of peripheral arterial disease and medial arterial calcification in patients with chronic renal failure: requirements for diagnostics. Am J Kidney Dis.

[2] WINSOR T (1950). Influence of arterial disease on the systolic blood pressure gradients of the extremity. Am J Med Sci.

[3] Chen SC, Chang JM, Hwang SJ (2010). Ankle brachial index as a predictor for mortality in patients with chronic kidney disease and undergoing haemodialysis. Nephrology.

[4] Ono K, Tsuchida A, Kawai H (2003). Ankle-brachial blood pressure index predicts all-cause and cardiovascular mortality in hemodialysis patients. J Am Soc Nephrol.

[5] Chen HY, Wei F, Wang LH (2017). Abnormal ankle-brachial index and risk of cardiovascular or all-cause mortality in patients with chronic kidney disease: a meta-analysis. J Nephrol.

[6] Zheng L, Li J, Hu D (2010). Association of low ankle-brachial index with mortality in patients with ischemic heart disease. J Atheroscler Thromb.

[7] Hyun S, Forbang NI, Allison MA (2014). Ankle-brachial index, toe-brachial index, and cardiovascular mortality in persons with and without diabetes mellitus. J Vasc Surg.

[8] Violi F, Davì G, Proietti M (2016). Ankle-Brachial Index and cardiovascular events in atrial fibrillation. The ARAPACIS Study. Thromb Haemost.

[9] Miguel JB, Matos JPS, Lugon JR (2017). Ankle-Brachial Index as a Predictor of Mortality in Hemodialysis: A 5-Year Cohort Study. Arq Bras Cardio.

[10] Caso V, Agnelli G, Alberti A (2012). High diastolic blood pressure is a risk factor for in-hospital mortality in complete MCA stroke patients. Neuro Sci.

[11] Protogerou AD, Safar ME, Iaria P (2007). Diastolic blood pressure and mortality in the elderly with cardiovascular disease. Hypertension.

[12] Rahman F, Al Rifai M, Blaha MJ (2017). Relation of Diastolic Blood Pressure and Coronary Artery Calcium to Coronary Events and Outcomes (From the Multi-Ethnic Study of Atherosclerosis). Am J Cardiol.

[13] Navaneethan SD, Schold JD, Jolly SE (2017). Blood pressure parameters are associated with all-cause and cause-specific mortality in chronic kidney disease. Kidney Int.

[14] Protogerou AD, Vlachopoulos C, Thomas F (2017). Longitudinal Changes in Mean and Pulse Pressure, and All-Cause Mortality: Data From 71,629 Untreated Normotensive Individuals. Am J Hypertens.

[15] Domanski MJ, Mitchell GF, Norman JE (1999). Independent prognostic information provided by sphygmomanometrically determined pulse pressure and mean arterial pressure in patients with left ventricular dysfunction. J Am Coll Cardiol.

[16] Hsu PC, Lee WH, Chen YC (2020). Comparison of different ankle-brachial indices in the prediction of overall and cardiovascular mortality. Atherosclerosis.

[17] Yamashina A, Tomiyama H, Takeda K (2002). Validity, reproducibility, and clinical significance of noninvasive brachial-ankle pulse wave velocity measurement. Hypertens Res.

[18] Messerli FH, Mancia G, Conti CR (2006). Dogma disputed: can aggressively lowering blood pressure in hypertensive patients with coronary artery disease be dangerous?. Ann Intern Med.

[19] Ikonomidis I, Makavos G, Lekakis J (2015). Arterial stiffness and coronary artery disease. Curr Opin Cardiol.

[20] McEvoy JW, Chen Y, Rawlings A (2016). Diastolic Blood Pressure, Subclinical Myocardial Damage, and Cardiac Events. Implications for Blood Pressure Control. J Am Coll Cardiol.

[21] Farnett L, Mulrow CD, Linn WD (1991). The J-curve phenomenon and the treatment of hypertension. Is there a point beyond which pressure reduction is dangerous?. JAMA.

[22] Tuomilehto J, Ryynanen OP, Koistinen A (1998). Low diastolic blood pressure and mortality in a population-based cohort of 16913 hypertensive patients in North Karelia, Finland. J Hypertens.

[23] Flint AC, Conell C, Ren X (2019). Effect of Systolic and Diastolic Blood Pressure on Cardiovascular Outcomes. N Engl J Med.

[24] Beddhu S, Chertow GM, Cheung AK (2018). Influence of Baseline Diastolic Blood Pressure on Effects of Intensive Compared With Standard Blood Pressure Control. Circulation.

[25] Vedel AG, Holmgaard F, Rasmussen LS (2016). Perfusion Pressure Cerebral Infarct (PPCI) trial - the importance of mean arterial pressure during cardiopulmonary bypass to prevent cerebral complications after cardiac surgery: study protocol for a randomised controlled trial. Trials.

